# Solidarity with whom? Minority perspectives on allyship in Danish queer spaces

**DOI:** 10.1111/bjso.12780

**Published:** 2024-06-28

**Authors:** Bao‐Thi Van Cong, Séamus A. Power, Thomas A. Morton

**Affiliations:** ^1^ Department of Psychology University of Copenhagen København Denmark

**Keywords:** allies, intergroup relations, intersectionality, social change, solidarity

## Abstract

Social psychological research has witnessed a burgeoning interest in advantaged group allies acting in solidarity with disadvantaged groups to challenge systems of inequality. While solidarity from advantaged group members is often deemed critical for social change, the perceptions of disadvantaged group members regarding ally participation are seldom addressed. This research delved into how LGBTQIA+ individuals in Denmark conceptualize allyship. Through 26 semi‐structured interviews with participants and organizers of queer pride events, a thematic analysis identified three themes addressing how allyship materializes, what risks it bears and who it involves. Specifically, we present a three‐levelled framework of allyship, which captures practices of allyship on a personal, relational and structural level. Our analysis also reveals the risk of allyship when it is not perceived as genuine and complexities of group boundaries when discussing allyship, shedding light on intersectional challenges within minority communities. These findings illustrate the nuances involved in providing and receiving allyship within and across various social (sub)groups.

## INTRODUCTION

The involvement of individuals from dominant and privileged groups—often called ‘allies’—working alongside members of marginalized and disadvantaged communities to pursue social change has been a recurrent theme in the history of social movements. For example, during the civil rights movement in the 1950s and 1960s, White allies stood beside African American activists in marches, protests and acts of civil disobedience, demanding an end to racial segregation and inequality. In the suffrage movement for women's rights, male politicians and activists supported women in the decades‐long fight to establish their right to vote in many parts of the world. Also in the United States, organizations like PFLAG (originally *P*arents and *F*riends of *L*esbians *A*nd *G*ays) have broadened their membership beyond individuals with direct relational bonds (i.e. parents and friends) to include a wider range of straight allies working to promote safety, equality and tolerance for sexual and gender minorities. More recently and in Europe, Estonian LGBTQIA+ activists were joined by heterosexual and cisgender allies in petitions and protests to legalize equal marriage. The Estonian parliament became the first of former Soviet states to pass a law supporting equal marriage in June 2023. These historical examples underscore the role that privileged groups can play in supporting marginalized groups in the fight for social justice. Indeed, while support for change might be less likely within privileged groups, some would say that it is essential since social change requires a redistribution of both material and symbolic power and privilege (Shnabel & Ullrich, 2013).

Social psychological research has greatly contributed to understanding the antecedents and consequences of collective action in support of social change (e.g. Becker & Tausch, 2015). Yet, despite a growing interest in the involvement and impact of advantaged/majority group members in social movements (Kutlaca, Radke, et al., 2020), perspectives on allyship among individuals from disadvantaged/minority groups are rarely considered. In this paper, we explore allyship from the perspective of the minority, more specifically, lesbian, gay, bisexual, transgender, queer/questioning, intersex, asexual/aromantic/agender and other sexual and gender minority (LGBTQIA+; in the following also collectively reported as queer) communities in Denmark. Through a qualitative investigation of how queer people in Denmark conceptualize allyship, we shed light on how allyship materializes, what risks it bears and who it involves.

### Social psychological predictors of action in support of social change

The social psychological analysis of social change movements has typically focused on the processes that motivate disadvantaged group members to challenge systems of inequality (for a research synthesis, see Agostini & van Zomeren, 2021). Integrating social identity theory (Tajfel, 1978; Tajfel & Turner, 1979) with concepts of relative deprivation (Power et al., 2020; Stouffer et al., 1949; Walker & Pettigrew, 1984) and self‐efficacy (Bandura, 2000), the Social Identity Model of Collective Action (SIMCA; van Zomeren et al., 2008) highlights the interrelated, but uniquely predictive, role of processes related to identity (e.g. Drury & Reicher, 1999, 2000), perceived injustice, particularly the emotional experience of anger (e.g. Miller et al., 2009; Tausch & Becker, 2013) and efficacy (e.g. Corcoran et al., 2011; Mummendey et al., 1999) in driving collective action to improve one's group's relative position within society. This model is supported by research drawing on a variety of data forms (e.g. Haugestad et al., 2021; Thomas et al., 2012, 2020) and cultural contexts (e.g. Cakal et al., 2011; Tabri & Conway, 2011), as well as meta‐analytic integrations (Agostini & van Zomeren, 2021; van Zomeren et al., 2008). More recently, SIMCA has undergone an extension to include moral motivations—that is, based on people's values, beliefs, principles and ideologies—as a further driving force behind collective action (Agostini & van Zomeren, 2021). Taken together, theory and research suggests that people engage in collective action if they strongly identify with a relevant social group, experience emotional reactions to perceived injustices or moral violations connected to this group, and believe that this group can achieve its goals.

Within this literature, a more recent, but growing, interest has been in the motivations of advantaged group allies to act in solidarity with disadvantaged outgroups (Adra et al., 2020; Radke et al., 2020; Uysal et al., 2022). Here, allies are typically understood as advantaged group members who hold affirming attitudes towards the disadvantaged group and who engage in action to combat group‐based inequalities (Brown & Ostrove, 2013; Ostrove & Brown, 2018). Meta‐analytic findings suggest the motivations for collective action proposed by SIMCA also explain advantaged group members' solidarity‐based actions (Agostini & van Zomeren, 2021), though not without caveats. For example, the role of identification—and the relevant targets of this—in guiding majority group collective action in support of minorities seems more mixed than is the case for minorities themselves (e.g. Górska et al., 2020; Osborne et al., 2019; Selvanathan et al., 2018; van Zomeren et al., 2011). Nonetheless, work that has investigated predictors of advantaged group members' engagement with social change suggests their motivations broadly overlap with those of disadvantaged group members.

### Other perspectives on majority allyship

One assumption that underlies research on allyship is that the involvement of advantaged group members in movements for social change is both desirable and important (Selvanathan et al., 2020). However, few studies to date have delved into the implications of allyship for disadvantaged groups, or into how involvement of majority allies affects the perception and reception of social change movements (Power, 2020). Some studies show that allies can have positive effects on the public perceptions of social change movements (Kutlaca et al., 2022), are effective in confronting biases within the majority (Chu & Ashburn‐Nardo, 2022; Gulker et al., 2013) and that this can contribute to well‐being among minority group members (Chen et al., 2023). But allyship has also been argued to sometimes harm rather than support social movements and the minority groups invested in these. For example, drawing on arguments about the ‘ironic’ effects of intergroup contact (for a meta‐analysis, see Reimer & Sengupta, 2023), Droogendyk et al. (2016) argue that positive contact between advantaged and disadvantaged group members may cultivate positive, trusting orientations towards the majority rather than the necessary feelings of anger and injustice that drive group‐based action to challenge systems of oppression.

In contrast to members of disadvantaged groups, the motives of majority group allies are more ambiguous. While some allies might have a genuine moral motivation for seeking social changes that dismantle the privileges of their own group, allyship can also be ‘performative’ with the goal of obtaining personal benefits, such as recognition and approval from others in their social context (Kutlaca & Radke, 2023). The ambiguity surrounding ally motivations may raise suspicions among its supposed beneficiaries (Kunstman et al., 2016; Marshburn et al., 2021) and result in perceptions of insincerity (Burns & Granz, 2023). Because advantaged group members are also perceived as having less understanding of issues connected to inequality, the visibility of majority allies as leaders in social justice efforts can deter participation by minority group members (Iyer & Achia, 2021). Involvement of allies further blurs intergroup distinctions, something that might be especially threatening to highly identified disadvantaged group members (e.g. Hasan‐Aslih et al., 2020). Finally, in addition to any identity concerns raised by allyship, research also suggests that the participation of allies may not have immediate instrumental value. Experimental studies find that ally involvement does not influence the readiness of authorities and decision‐makers to comply with movement demands (Hartwich et al., 2023).

### Intraminority solidarity

Apart from advantaged group members acting in solidarity with disadvantaged outgroups, solidarity has also been studied between members of different marginalized groups. Based on the common ingroup identity model (Gaertner et al., 1993; Gaertner & Dovidio, 2000), research suggests that perceiving a shared destiny is linked to an interest in forming coalitions between members of differently marginalized groups (i.e. stigma‐based solidarity; Chaney & Forbes, 2022; Craig & Richeson, 2016). This applies for groups that are marginalized along the same identity dimension, for example, between Black and Asian Americans (Craig & Richeson, 2012), but not necessarily for groups marginalized along differing identity dimensions, such as Black Americans and LGBTQIA+ people (Craig & Richeson, 2014). The concept of critical consciousness has also been introduced to better understand and explain patterns of intraminority solidarity (Burson & Godfrey, 2020). Critical consciousness is theorized to involve three components: critical reflection on the historically underlying causes of oppression; political efficacy in the form of perceived ability to contribute to socio‐political change; and critical action, both individual and collective, to address societal injustices. Critical consciousness is argued to feed into similarity and common identity between different minority groups, while attenuating competition, in ways that promote intraminority solidarity.

Though theorized and studied in a variety of group contexts, most work on solidarity still focuses on singular dimensions of (dis)advantage. Intersectional perspectives instead highlight how various forms of inequality, such as race, gender, sexuality and class, can interact and affect individual opportunities and experiences in ways that are not reducible to single dimensions alone (Collins, 2022; Crenshaw, 1989). Taking this up, some recent research has considered how multiple identity dimensions interplay to determine solidarity‐based collective action (Uysal et al., 2022), confrontation (Case et al., 2020) and support for intraminority coalitions (Pham et al., 2023). Growing awareness of intersectionality in the solidarity literature raises additional, and more complex, questions about relations between subgroups within a minority that may be more or less privileged based on the additional identities they possess, for example, between LGBTQIA+ people with and without disabilities. Yet, despite awareness, the issue of intersectionality as it relates to solidarity across subgroups within a single minority remains to be fully explored.

### Conceptual challenges

The above review suggests the assumed value of majority allies in social movements towards equality is not unequivocal. In addition, the analysis of allies' role in social movements has yet to be fully explored from an intersectional perspective, which complicates neat dichotomies of advantage–disadvantage. Given this, it would seem important to delve more deeply into exactly how allyship is understood, and the different meanings and motives that may be attached to this concept from different perspectives—not just across majority and minority groups but also within them. Yet, despite increasing scholarship on allyship, the concept remains less than fully defined and varying interpretations are deployed across studies (Kutlaca, Radke, et al., 2020; Louis et al., 2019). Researchers have employed diverse terminology to describe both the concept itself—including solidarity, political solidarity, collective action on behalf of marginalized groups, allyship and ally activism – and the behaviours through which it may be revealed—ranging from supportive interpersonal interactions to political activities like participating in protests in support of disadvantaged communities (Louis et al., 2019). An additional challenge in defining allyship lies in its dual usage, encompassing not just individual and collective actions but also the underlying motivations (Kutlaca, Radke, et al., 2020). In sum, allyship is a concept that is used to describe an array of motivations and behaviours related to intergroup prosociality with the primary focus to date on the advantaged group.

Lack of conceptual clarity has been identified as a pervasive and long‐standing issue within the field of psychological science, which interferes with researchers' ability to meaningfully build, interpret and apply theories and models (Bringmann et al., 2022). Conceptual unclarity therefore undermines theory‐driven research. Recommendations for improving conceptual clarity include, among others, going back to the basics of conceptualization through re‐evaluating the phenomena at stake (Rozin, 2001). Here, qualitative research can offer a breadth of information and insights that may be lost when researchers move too quickly to quantification. Opening concepts up through qualitative inquiry can help researchers to better understand how those involved in a focal phenomenon understand and experience the concept in question (Bringmann et al., 2022; Power et al., 2018).

In this spirit, this study responds to the growing scholarly interest, but persistent conceptual fuzziness, in ‘allyship’ by engaging in a qualitative examination of minority individuals' own conceptualizations of this phenomenon. The specific context framing this investigation is social change in support of LGBTQIA+ identities. This is a context in which popular discussions of allyship are alive, but which is also relatively understudied within the social psychological research on allyship, which to date has focused mostly on racial and traditional gender dynamics (e.g. Chu & Ashburn‐Nardo, 2022; Kutlaca, Becker & Radke, 2020; Kutlaca, Radke, et al., 2020). We conducted this research among members of queer communities in Denmark, specifically in Copenhagen. Given its specificity, some background to this context is warranted.

### The Danish context

As the first country in the world to legalize same‐sex registered partnerships in 1989 (Bech, 2006), Denmark is often recognized as one of the most progressive countries in relation to LGBTQIA+ rights. In the 2023 Rainbow Europe Index of the European International Lesbian, Gay, Bisexual, Trans and Intersex Association (ILGA‐Europe), which evaluates 49 European countries based on their respect for the rights of LGBTI people, Denmark ranked third, highlighting its position as one of the leading countries in Europe in respecting and protecting the rights of its queer communities (ILGA‐Europe, 2023). It has been argued that issues related to homosexuality have become mainstream in Denmark and that, legally speaking, only details are left to achieve equal rights (Edelberg, 2014). Sociologist Henning Bech (2006) would go so far as to argue that homosexual, gay, lesbian and queer *identities* are disappearing, and are now instead thought and spoken of as sexual *tastes*. This is linked, among others, to the Danish welfare state and its associated assurance of basic levels of financial and social security, providing the conditions for ‘non‐traditional’ lifestyles. Bech (2006) also explains that the cultural ideology of ‘frisind’, literally translated as ‘free mind’ or ‘free spirit’, which is widely lived by in Denmark, encourages people not only to live according to their own preferences (i.e. tastes) but also to commit to provide the conditions for others to do the same. Despite this progressive history, and the apparent ‘mainstreaming of gender and sexuality’ (Ilmonen et al., 2017, p. 99) in Denmark, the Danish police's annual report (Nebeling Petersen, 2023) shows that the number of hate crimes against LGBTQIA+ people are increasing. Accordingly, present‐day Danish queer activism targets hate crimes and social rather than legal discrimination (Edelberg, 2014).

Denmark's largest LGBTQIA+ event, Pride Week, is organized by Copenhagen Pride with over 100 events, ranging from debates and workshops to concerts, ending with its popular parade. According to the organization, more than 30,000 people participated in the parade in 2022 and an estimated 250,000 people came to stand and watch along the route (Copenhagen Pride, 2022). In 2022, for the second time, Fluid Festival organized a separate space for women, genderqueer and non‐binary identities temporally embedded in Pride Week (Fluid Festival, 2023). This four‐day festival offered talks, debates, and entertainment from and for female‐identified, gender non‐conforming, and non‐binary people. Since 2018, Nørrebro Pride has been organized as a non‐commercial, anti‐racist and intersectional alternative to Copenhagen Pride, centering Queer, Trans, Intersex, Black, Indigenous People of Color (QTIBIPOC; LGBT+ Danmark, 2022). This event has attracted considerable media attention due to, for example, the division of demonstrations into blocks consisting of a maximum of 50 people, with the front of the march exclusively reserved for QTIBIPOC (Stisen, 2021). Some media commentators (e.g. Sindberg, 2021) have argued that Nørrebro Pride's strategy of racial segregation might perpetuate the very problem the organization is protesting against. At the same time, ethnographic fieldwork surrounding Nørrebro Pride has shown its importance in publicly demonstrating the unsustainable conditions for life of the local but also global QTIBIPOC communities as well as in fostering agency to assemble collectively as a local community (Burø et al., 2024).

We found the context of Pride, and the various sub‐ and splinter events attached to it, a particularly interesting one to study. All events are part of the same broad social movement concerned with addressing discrimination and improving the position of LGBTQIA+ individuals in Danish society. However, different events intersect this focus with concern for additional gender and racial identities, reflecting the growing engagement with intersectional dynamics in queer communities. The events, and the organizations they represent, also offer differing positions on who is and is not welcome in queer spaces. We reasoned that sampling across these events could offer a range of insights into whether and what type of allyship is needed according to members of the communities, as well as potential intersectional perspectives on allyship.

### This research

The central objective of our study was to examine the ways in which people in queer communities in Denmark conceptualize allyship. More specifically, we aimed to identify, analyse and expand on queer people's understandings of the role of allies in Danish queer spaces, both social and physical. Due to our interest in the minority perspective, we adopted an emic approach in that we focused specifically on the conceptual definitions and interpretations of members of our community of interest. In doing so, we intended to unravel the cultural meaning and significance of allies and allyship in general as understood by community members. To this end, we conducted semi‐structured interviews to arrive at thick descriptions (Geertz, 1973) of allies' characteristics, behaviours and functions to help shed light on the nuances of allyship in Danish queer spaces.

## METHOD

### Participants

We conducted semi‐structured interviews (*N* = 26) from August until November 2022 with participants who either attended or were involved in organizing either one or multiple of the events offered by Copenhagen Pride, Fluid Festival or Nørrebro Pride. Participants who attended Copenhagen Pride or Fluid Festival were approached and interviewed on the events' premises. Co‐organizers of these events were contacted via email and interviewed at a place of their choice, usually their office. As Nørrebro Pride organized separatist assemblies aimed at creating a safer space for QTIBIPOC, we intentionally chose not to participate in their events as researchers. Instead, we recruited participants via personal contacts and a Facebook group for queer people in Denmark based on whether they had attended events hosted by the organization. Some of these interviews took place on university grounds, others at a place chosen by participants. We concluded data collection following considerations of various aspects, such as the depth, breadth, complexity and general quality of the data (Braun & Clarke, 2021).

Participants were a mix of organizers, long‐standing activists and attendees of Pride events and the broader LGBTQIA+ movement in Denmark. All participants interviewed in relation to Nørrebro Pride (*n* = 6) were either first‐ or second‐generation immigrants to Denmark of different ethnic backgrounds, while those interviewed in relation to the other two organizations, which do not specifically focus on ethnic minorities within LGBTQIA+ communities, were a mix of nationals and internationals. All participants identified as one or multiple parts of the LGBTQIA+ acronym and were aged between 19 and 60 years (*M* = 29.78, *SD* = 9.87).

### Materials and procedure

We obtained oral consent from the respondents before conducting the interviews. All interviews were audio recorded and lasted between 2 and 38 min. Short interviews were carried out with participants who were approached while at the event (e.g. at the Pride parade or at a panel discussion; *n* = 14) and longer interviews with those with scheduled appointments (*n* = 12). This combination of short *in situ* interviews and longer retrospective interviews was chosen to ensure both breadth and depth of data. On 1 day of data collection on the premises of Copenhagen Pride, the first author was joined by a colleague to increase the amount of data to be collected. The colleague, who is a trained qualitative researcher, was familiar with the interview guide and conducted three of the *in situ* interviews included in this study. All other interviews were conducted by the first author. The interviews were held in English and participation was voluntary and not compensated.

After introducing herself and obtaining verbal consent, the interviewer went through a semi‐structured interview guide. At the start of the interview, participants were asked whether they attended or were planning to attend other Pride events and what they hope to achieve with participating in (in the case of participants) or co‐organizing (in the case of organizers) such events. These first two questions aimed to ease participants into the interview and explore their prior experience and engagement with Pride‐related events as well as their motivations and intentions related to these events. We then probed interviewees to explore questions of inclusivity and exclusivity by asking, ‘Who is and is not welcome at this event?’, followed by the question of why this matters. This question was also intended to get participants to talk about allies without being explicitly suggestive or leading. Many participants already mentioned allies at this point in the interview. In this case, the interviewer followed up by asking, ‘What is an ally to you?’ or ‘What does allyship mean to you’, depending on whether allies or allyship was mentioned. In the case of no mention of allies or allyship, this question was brought up by the interviewer herself. Participants were then asked to reflect on what it means to be a good ally, and more specifically, how these act and what they do. If participants seemed to find it difficult to answer this abstract question, the interviewer introduced a hypothetical person, a cisgender heterosexual man who would like to support the LGBTQIA+ movement. The interviewer then asked, ‘What should he do?’ and ‘What should he not do?’. At the end of the interview, all participants were asked whether they identified as one or more of the identities related to LGBTQIA+ and for their age, except when they already answered this during the interview. After the interview, participants had the chance to ask questions to the interviewer. All participants, no matter whether they were interviewed as organizers or participants of a pride event and whether they were interviewed *in situ* or in retrospect, were asked the questions above. As usual in semi‐structured interviews (Braun & Clarke, 2022), follow‐up questions varied according to what participants were sharing. Longer, retrospective interviews allowed for more follow‐up questions, while shorter, *in situ* interviews usually closely followed the interview guide.

### Analytic approach

The interviews were transcribed verbatim. The analysis of the qualitative data followed a process in line with the principles of inductive thematic analysis (Braun & Clarke, 2006, 2022) using NVivo. This approach allowed for a systematic exploration of the interview data to identify patterns and themes across participants' narratives. Our analytical procedure was underpinned by a critical realist and contextualist perspective, a framework that recognizes that any external, objective reality can only be observed through the lens of sociocultural meanings and the interpretative resources of the researchers and participants embedded in their social context. This approach enabled us to explore the complex interplay between individual experiences and socio‐cultural contexts in shaping perceptions of allyship within LGBTQIA+ communities.

The first author performed the analysis by carefully reading the interview transcripts and scanning them for relevant comments and patterns of meaning. Interesting features were systematically coded across the transcripts and grouped into themes. When necessary, the transcripts were revisited to ensure proper representation and contextualization of the data. The themes were further collated into meta‐themes, eventually generating a preliminary thematic framework. After discussing the preliminary themes within and outside of the research team, we refined and labelled all themes and subthemes. We reorganized and renamed themes several times until the final thematic framework was developed.

We engaged in individual and collective reflexivity throughout the research process. Recognizing the potential limits of our perspectives as non‐natives to the socio‐cultural landscape of Denmark, we consulted a Danish researcher specialized in Scandinavian LGBT history at the beginning of our project who provided valuable input to our research ideas and recruitment plan. As our research team comprised individuals with diverse sexual orientations and ethnic backgrounds, we reflected on and discussed our status as both insiders and outsiders to the studied communities at multiple stages of the research. For example, during interviews with queer people of colour, the first author noticed that interviewees appeared to establish stronger rapport with her. Due to the first author's phenotypically evident migratory background and her membership in a Facebook group for queer people in Denmark, where the study was advertised, she was likely perceived as a member of the ingroup, resulting in a more pronounced sense of connection and relatability. Some interviewees of colour also inquired about the interviewer's specific identity in both implicit and explicit ways. Interestingly, White interviewees seemed less concerned with the interviewer's position within queer communities in Copenhagen, perhaps reflecting the relative higher social acceptance and sense of safety experienced by White LGBTQIA+ individuals in Denmark. It should be noted that insider/outsider boundaries are not always clear cut, nor are they mutually exclusive categories (Hayfield & Huxley, 2015). Since both insider and outsider perspectives come with benefits and challenges, we engaged in ongoing reflections and discussions on the complex, intersecting identities and positions we hold in relation to our research participants.

## RESULTS

We report three themes in this article. ‘Levels of allyship’ captures a framework through which to understand the forms of allyship presented by interviewees, while ‘Performative allyship’ addresses the potential negative consequences of allyship that is not perceived as genuine. ‘Negotiation of group boundaries’ reveals the complex identity dynamics in LGBTQIA+ communities that accentuate power imbalances between subgroups of communities and how queer people navigate that social space. This highlights the importance of enacting allyship within and across subgroups of LGBTQIA+ communities, especially for intersectionally marginalized groups. In presenting these themes, we address questions on the manifestations, risks and groups involved in allyship (Table 1).

**TABLE 1:** Thematic overview.

Themes	Subthemes
Levels of allyship	Personal
	Relational
	Structural
Performative allyship	
Negotiation of group boundaries	Intersectional safe(r) space
	Ingroup allyship
	Intergroup allyship

### Theme 1: Levels of allyship

Participants discussed their experiences and perceptions of allyship across multiple levels, including personal, relational and structural dimensions. From this, we identified a multi‐level framework capturing the different accounts of allyship practices and the contexts in which they take place. Organizing the mentioned practices into this multi‐level structure reveals the complex nature of allyship, which encompasses not only cultivating personal development but also challenging the broader socio‐political context.

#### Personal: ‘Actively working with your own biases’

At the personal level of allyship, some interviewees underscored the importance of being willing to investigate, (un)learn and consider one's positionality within or in relation to LGBTQIA+ communities. Participants highlighted the significance of taking proactive steps to educate oneself about LGBTQIA+ issues, actively challenging one's own biases, and engaging in self‐reflection.

‘What he [an ally] can do is to get informed, to get, um, to get himself, uh, to get knowledge, to learn and probably not to go around and ask people who are, like, if they see someone dressed up and be like, “Oh, let me ask everything”. Because like they don't have the energy. They do that all the time. They don't owe him anything’. (Interviewee 23)

As interviewee 23 explains, engaging with allyship on the personal level can relieve LGBTQIA+ individuals of the burden to act as a representative and educate others about queer issues. Actively working with oneself serves as the basis of allyship, which practices at other levels build upon. Individuals who label themselves as allies, but who are not involved in allyship at a personal level, can be perceived negatively (see *Performative allyship*). In line with previous findings on the importance of allies' commitment to lifelong learning (Selvanathan et al., 2023), interviewees in our study highlighted that allyship begins with oneself, going inward and committing to ongoing learning, ultimately enhancing the authenticity and impact of allyship efforts.

#### Relational: ‘A good ally will speak up for me when I'm not there’

The relational level of allyship uncovers the significance of interpersonal actions that impact the experiences of LGBTQIA+ individuals within their social networks. Participants especially emphasized the importance of allies speaking up and advocating for LGBTQIA+ rights in various contexts, including within their families, peer groups and in public spaces. Respondents described actions involving challenging prejudiced attitudes and behaviours as well as intervening in instances of discrimination.

‘I see a lot of allyship being very silent, very like, “I support you, but…”. But then, you know, when somebody says something, uh, rude, for example, to me or a friend (I: mh hm) or something racist or something transphobic or homophobic (I: mh hm), then, um, it's sort of like afterwards, they can say, ‘Oh, that was really… What a dickhead’, but, um, but in the actual scene when it plays out (I: mh hm), they don't say anything (I: mh hm) and you're left to fend for yourself’. (Interviewee 7)

This interviewee reflects on the gap between intention and behaviour when it comes to allyship. Previous research on intergroup relations and prejudice reduction has highlighted the critical impact of allies confronting biases on minority group members' self‐esteem and perceptions of allies (Chu & Ashburn‐Nardo, 2022; Kutlaca, Becker & Radke, 2020). Interviewees in our study emphasized that speaking up for LGTQIA+ individuals can contribute to safer and more comfortable social environments.

Participants most frequently discussed allyship practices on the relational level. The emphasis on relational allyship in these discussions can be seen as a reflection of the current landscape of LGBTQIA+ activism in Denmark. It highlights the recognition that, while many legal victories have been achieved (Edelberg, 2014), the work of dismantling deeply ingrained societal biases and prejudices also requires active engagement at the interpersonal level. In this context, interviewees perceived relational allyship as a crucial tool for advocating for LGBTQIA+ individuals. At the same time, scholars have critiqued some of these actions as ‘individualized remedies’ that provide a mechanism through which inequality is maintained and justified (Sumerau et al., 2021). To understand the nuances and impact of allyship, it is therefore important to acknowledge the dynamic interplay of forms and levels of allyship and their effect as contingent upon the ever‐evolving socio‐political context.

#### Structural: ‘Pushing for systemic changes’

At the structural level of allyship, interviewees remarked that practicing allyship necessitates actions that transcend individual interactions and extend into the realm of systemic change. Participants expressed the belief that effective allyship involves not only providing personal support but also actively engaging in advocacy and activism to challenge and transform existing structures of inequality. These actions can include advocating for LGBTQIA+ rights in policy and legislation, participating in community organizing and protests, providing financial resources to LGTBQIA+ organizations and pushing for inclusivity within institutions and workplaces.

When reflecting on the role of allyship in activism, several participants emphasized the critical importance of allyship involving a thoughtful consideration of the space one occupies within queer spaces. In accordance with previous research on disadvantaged group members' attitudes and perspectives on allyship (Park, 2022; Selvanathan et al., 2023), interviewees highlighted the significance of allies being mindful of their presence and not overpowering or dominating conversations and actions. This notion of ‘supporting from the back’ resonates with the idea that effective allyship often requires humility, listening and providing support without taking the spotlight.

Importantly, one participant underscored the need to acknowledge and embrace the diversity and differences that exist within any social movement or community:

‘I think it's really important within a movement, as in the people who are part of that, that we can always remember that even if we come together as a movement, we won't all have the same struggles (I: mh hm). So within the movement, we need to like remember to, um, struggle with these differences, across these differences, discuss them, agree on where to go maybe, maybe disagree sometimes, come back together (I: mh hm). And maybe that could be something that could also be thought of as forms of allyship within a movement or within a community (I: mh hm)’. (Interviewee 10)

As respondent 10 notes, LGBTQIA+ communities are not monolithic and encompass individuals with varied experiences, identities and needs. Effective allyship, therefore, involves recognizing and respecting these differences.

The structural dimension of allyship underscores the role of allies as agents of broader societal change and aligns with the contemporary research focus on allies using their privilege to dismantle systemic barriers (e.g. Kutlaca, Radke, et al., 2020). Our findings resonate with this body of research by highlighting how allyship, from both within and outside of LGBTQIA+ communities, can support processes of change at a structural level. Participants in this study underscore the importance of how allies engage with the movement, for example, not exerting dominance in their interactions and actions.

### Theme 2: Performative allyship

Some interviewees reflected on potential risks of allyship being perceived as performative, particularly when individuals self‐label as allies and it becomes a dominant aspect of their identity. Interviewees voiced concerns that when allyship is publicly declared and overly emphasized as a personal identity, it can come across as insincere or superficial. They pointed out that performative allyship may prioritize appearances over genuine support, potentially overshadowing the authentic solidarity that LGBTQIA+ individuals seek from allies. As participant 12 describes:

‘People might say they're an ally or people might, you know, claim the title of ally themselves and then still really misunderstand a lot of things (I: mh hm) about the community and also about identities in general or sexualities. Um, and in that case, I guess I'm not saying they can't be an ally, but perhaps they're not really educated (I: mh hm) about everything and that feels iffy to me (I: mh hm)’. (Interviewee 12)

This participant seems to be pointing mostly to the lack of personal‐level allyship as an indicator of performative allyship, while other respondents suggested that engaging on multiple levels of allyship may be necessary to move beyond performative allyship. For example, interviewee 21 notes that it takes more than attending pride to be considered an ally at all: ‘If you just show up to a few Pride parades, you're not really an ally. You don't do any activism on top of your performances’.

In current social psychological literature, performative allyship is understood as a type of allyship including action that requires little effort, is motivated by personal needs and is primarily driven by self‐interest (Kutlaca & Radke, 2023). Allyship that is perceived as more costly (socially, occupationally or financially) has been shown to be evaluated more positively compared to allyship associated with rewards (Thai & Nylund, 2024). Overall, evidence suggests that engagement in performative allyship by members of advantaged groups can have adverse effects on disadvantaged group members, especially those who are highly observant and attuned to such inauthentic involvement (Kutlaca & Radke, 2023).

Participant 10 further reflects on the issue of allyship as an identity:

‘I'm not sure if I would say anyone is an ally as like a form of being but more like he definitely practices forms of allyship for me, for example, and then I practice forms of allyship for others, but I wouldn't claim it as an identity or something’. (Interviewee 10)

As this interviewee explains, conceptualizing allyship as a practice rather than identity holds the potential to resolve the tension related to performative allyship. If performative allyship is seen as a result of centring oneself and over‐identification as *an ally*, focusing on practices may shift the attention from the self to the persistent process of *allyship*.

In most research, allies are studied as a kind of people (e.g. Johnson & Pietri, 2022; Kutlaca, Radke, et al., 2020). As participant 10 describes, allyship is not solely confined to a distinct group of people who actively identify as such; rather, it is a fluid and evolving set of actions and behaviours that can be enacted by individuals across various social identities and contexts. This perspective challenges the assumption that allyship is solely an identity and underscores the need for a more comprehensive and inclusive understanding of allyship that recognizes its dynamic, situational nature. Ultimately, a shift to understanding allyship as a practice rather than an identity might minimize the dangers of performative allyship.

### Theme 3: Negotiation of group boundaries

When reflecting on allyship, participants in our study flexibly negotiated the boundaries of subgroups within LGBTQIA+ communities. One respondent explained that they can sometimes be an ally, while other times needing an ally. This theme underscores the significance of allyship as a dynamic force that operates within and between different (sub)groups of LGBTQIA+ communities, which particularly holds relevance for intersectionally marginalized communities. While some interviewees discussed allyship that addresses forms of discrimination or marginalization that are prevalent within queer communities, others sought a form of allyship that fosters acceptance and solidarity from the cisgender and heterosexual majority. Apart from support given by relatively advantaged individuals both within and outside of queer communities, respondents expressed the importance of intersectional safer spaces that focus on intersectional subgroups, such as racialized queer individuals, to support one another and centre the specific struggles and needs of this group. Challenging the notion of allyship as a practice delivered solely by individuals from advantaged positions in society, the relevance of intersectional safer spaces suggests that solidarity between those with similar minoritized identities holds particular meaning for intersectionally marginalized individuals. We used the term ‘safe(r)’ to acknowledge some respondents' original phrasing of ‘safe space’, while we chose the term ‘safer’ when discussing safer spaces, following existing research and theorizing suggesting that safety requires continuous negotiation and can never be warranted (Lohman, 2022).

#### Intersectional safe(r) space: ‘I always feel like I don't belong to other queer events’

Several interviewees shared their experiences of bearing the burden of discrimination not only based on their LGBTQIA+ identity but also their other characteristics, highlighting the complex interplay of multiple minoritized identities. These individuals expressed that having intersectionally marginalized identities often resulted in unique and layered experiences that were challenging for others to fully comprehend. Research indeed shows that individuals with intersecting minority identities can be confronted with distinctive challenges that relate to stress and trauma (Ching et al., 2018), mirroring the accounts of participants in our study.

For many, participating in spaces provided by organizations like Nørrebro Pride was an opportunity to practice solidarity within their intersectionally marginalized subgroup and be a part of a community where they could find understanding and address specific needs amidst the complex challenges they faced. Participant 11 reports:

‘I feel like for me it's important cause like if there's no space like this, I I always feel like I don't belong to the other queer events (I: mh hm). Because for me like queerness doesn't only mean I'm a non‐binary person or I'm a gay or something, it means like because of the political environment in China and I'm a people of color in Denmark, a very White society, it means much more, so like very intersectional concept. I feel like fighting for queerness also meaning fighting for my identity here (I: mh hm)’. (Interviewee 11)

LGBT spaces such as Copenhagen Pride have more recently been criticized for being ‘homonormative’, that is aligning political goals with heteronormative values related to neo‐liberalism, such as monogamy and commercialization (Duggan, 2002). This shift has arguably caused Pride events to stray from their radical political roots, leaning instead towards politics that prioritize conformity to the values and standards congruent with White and affluent perspectives (Kates & Belk, 2001). Pride events like this can exclude parts of LGBTQIA+ communities that do not resonate with homonormative discourses, especially queer people of colour and transgender people (Kenttamaa Squires, 2019). In this way, some contend the essence of safer spaces extends beyond mere safety, and that their role also crucially involves promoting interaction and cooperation among the occupants of such spaces (Bowstead, 2019).

While safer spaces within intersectionally marginalized subgroups can serve as vital sources of support and understanding for some, it is worth noting that not all participants in our study endorsed this strategy. Some participants raised concerns that an overemphasis on identity‐specific safer spaces could inadvertently lead to more exclusion and reinforce divisions within LGBTQIA+ communities. For others, such spaces raised doubts and insecurities about their own role in such subgroup identities:

‘The year before that, I remember sort of feeling a little bit left out of the community, the QTIBIPOC sort of community, um, at the time, um, especially being, you know, QTIBIPOC in the way that I am. I think I can feel very anxious already (I: mh) being in QTIBIPOC space and I should probably clarify that I'm half Thai and half Danish (I: mh), which means that I'm Wasian (I: mh hm), um *chuckles*, for lack of better words. So I often feel in QTIBIPOC spaces as if I'm taking up too much space (I: mh hm), as I'm still kind of White (I: mh hm), um, and I'm like, “Should I really be here? Maybe not. I don't know.” Cause my struggles are definitely not the same (I: mh hm) as someone who, I don't know, is Black or, um, Muslim or, uh, fully Asian or adopted (I: mh hm)’. (Interviewee 12)

These excerpts underscore the intricate process of crafting collaborative queer safer spaces, which involves challenges of balancing safety and queerness that are not unique to the Danish context. For example, McCartan and Nash (2022) examined the processes through which a grassroots LGBT Pride group in Scotland attempted to establish a radical, inclusive space primarily for transgender individuals but faced controversy over an initial (but later reversed) drag performer ban. Analysing an array of qualitative data, the authors conclude that creating queer safer spaces necessitates continuous engagement with the viewpoints of the marginalized groups they aim to support, focusing on their perceptions of what it means to feel safe, what it means to be queer and how these interact.

#### Ingroup allyship: ‘Sometimes I'm also an ally within the queer community towards struggles that are not my own’

Some participants discussed their role and responsibility in using their relative privilege within LGBTQIA+ communities to offer support to other, less privileged, members of the community.

‘When you're talking about allyship, you tend to talk about supporting groups that are in some kind of way faced with structural oppression (I: mh hm) and when you're part [of] the LGBTI+ community […], your kind of your starting point is that you kind of, in a very broad sense, know what discrimination feels like or […] to face barriers due to your sexual orientation or your gender identity or who you are. And I think you would think at least that that would make you a lot more sensitive to oppression that other people face, for instance, due to, um, physical disability, hidden disability (I: mh hm), um, minority ethnic, um, status, HIV status, you know, these kind of things that not everybody in the community, um – we talk a lot about double minoritized people (I: mh), um, unfortunately realized that's not, you know, always what it looks like. But I think when you're being an ally while also needing allyship, I think, is an interesting position (I: yeah), because you know how important it is (I: mh hm), um, to have that support’. (Interviewee 25)

Respondent 25's reflections highlight the importance of recognizing that, even within marginalized communities, varying degrees of privilege exist, and with this privilege comes a responsibility to promote solidarity among community members. When asked to speak about their understanding on allyship, many respondents reflected on both their need for support as an LGBTQIA+ person as well as the need to support other LGBTQIA+ people with varying degrees of privilege along other dimensions of identity. Research suggests that being marginalized on multiple dimensions of one's identity predicts one's belief that prejudices co‐occur (i.e. Lay Theory of Generalized Prejudice) and, consequently, one's perception of similarity between the experiences of oppressed groups and willingness to engage in intraminority coalitions (Pham et al., 2023). In line with the reflections of respondents in this study, this suggests that intersectional disadvantage makes one more sensitive to the intersecting roots of prejudice, which unveils the importance of supporting one another across differing dimensions of oppression, highlighting the importance of intersectionality and positionality in intragroup solidarity.

At the same time, acknowledging and engaging with one's position within LGBTQIA+ communities can increase expectations towards those who hold relative privilege to actively advocate for and support those with intersecting marginalized identities. Some interviewees expressed their dissatisfaction with the engagement of White, gay men with issues outside of their own interest:

‘I think for me when I think of the word ally, because I have, uh, these different identities within the queer community for being, you know, queer, trans and Brown. And Brown is a very big part of my experience in the queer, uh, community and I think a lot of people don't, uh, count that, because that has nothing to do with my gender or sexuality but it's a really part of how I'm perceived in the community (I: mh hm). Um, so for me it's it's completely strung together. So when I think ally, I usually I think for me allyship between different parts of the community (I: mh). I think that's a really big part of allyship (I: yeah). And I think when I look at Pride, or when I look at public spaces even (I: mh hm) […], I think that, um, those are the allies that I think most about, how they can do things better (I: yeah). Um, and because that's where I'm usually the most disappointed as well if they don't do a good job. *chuckles* (I: mh hm). Um, so for me I would say even, you know, even in queer spaces, allyship can be a lot better’. (Interviewee 7)

This excerpt reveals how heightened expectations placed on relatively privileged members of the LGBTQIA+ community can lead to negative consequences, when these expectations are not met. Striking a balance between acknowledging privilege and navigating the demands it may bring is crucial to fostering a more inclusive and supportive community environment.

#### Intergroup allyship: ‘We do need straight allies to take on queer issues’

To some participants, allyship provided by the cisgender and heterosexual majority served its own purpose related to visibility and acceptance.

‘And at the same time, I think especially when we talk about struggles of people who are minoritized and marginalized parts of, uh, parts of the work needs to come from people who are not affected by these struggles, so allies in the sense of not being part of that community or that movement maybe necessarily (I: mh hm). So I think, you know, we do need straight allies to to take on queer issues and I think that's what we were saying before with like Copenhagen Pride. I know that it has an important purpose (I: mh hm). Um, because it's also a space for straight allies to join, um, and stand up for LGBT rights and so on and I think that's super important and we need White allies against racism’. (Interviewee 10)

Another interviewee further emphasized that straight allies, by virtue of their privileged status in society, possess a unique ability to advocate for and amplify LGBTQIA+ voices, as ‘Bigots and people who are intolerant don't listen to people in this community, but for some reason, they will listen to a straight cisgendered person when they say it’ (Interviewee 19). What interviewee 19 suggests in this excerpt resonates with observed patterns in the realm of sexism. Studies indicate that men who address sexist behaviours are often viewed in a more favourable light, and their interventions are deemed as more legitimate (Drury & Kaiser, 2014). Additionally, when men point out sexist behaviour, it is associated with heightened self‐confidence and reduced adherence to stereotypes in targets of sexism (Cihangir et al., 2014). This highlights the potential influence and responsibility of individuals outside minority groups in challenging discriminatory attitudes and fostering a more inclusive environment.

Overall, this theme illustrates that allyship is not confined to singular identities or specific groups; rather, it can span across the diverse landscape of LGBTQIA+ communities. Interviewees report that different subgroups within queer communities often engage in allyship with distinct purposes in mind. For instance, some may engage in allyship to address forms of discrimination or marginalization that are prevalent within their subgroup. Others may seek to leverage their relative privilege to foster solidarity between different subgroups. Importantly, these forms of allyship are not mutually exclusive, meaning that intersectional safer spaces can co‐exist with manifestations of intergroup allyship involving sexual and gender majorities.

## DISCUSSION

The last decade of social psychological research into social change has witnessed growing attention to advantaged group members and their involvement in the ongoing fight for more equitable societies. Yet, this scholarship has typically focused on the factors that drive individuals to act in solidarity with disadvantaged groups, inadvertently sidelining the perspectives of those they advocate for. With a focus on LGBTQIA+ communities in Denmark, we aimed to contribute to a clearer conceptualization of allyship by investigating how it is understood by those it intends to support. Examining how queer people in Denmark understand allyship, we identified three themes in participants' responses: the discussion of allyship on three interacting levels, the risk of performative allyship and the negotiation of group boundaries when identifying the relevant actors of allyship.

The proposed three‐levelled framework for allyship offers a more structured lens through which the prevailing conceptual ambiguity described by scholars in the field can be addressed (Kutlaca, Radke, et al., 2020; Louis et al., 2019). Mirroring findings from previous research on allyship (Brown & Ostrove, 2013; Selvanathan et al., 2023), this framework specifies tangible behaviours regarded as allyship by minority group members. While much of this earlier research has been rooted in racial and traditional gender contexts (e.g. Brown & Ostrove, 2013; Cihangir et al., 2014), our study contributes to this literature by extending this understanding to the realm of sexual and gender minorities. Notably, our study underscores that allyship is multifaceted, manifesting across varying levels and forms of action, sometimes interdependent in nature. The salience of each level and their interrelations might be influenced by the specific socio‐political context. In the Danish context, where LGBTQIA+ issues are more prevalent in social spheres, the personal and relational levels of allyship emerged as particularly significant. Practicing allyship on the structural level, however, was only perceived positively when seen as supportive rather than dominant. Beyond viewing allyship as an identity and allies as a group of people, our research additionally accentuates the importance of studying allyship as a *practice* to ensure a positive reception by the minority group in question.

A notable insight of our study lies in its engagement with the intersectional nuances present within queer communities. When discussing collective organizing surrounding social issues, our findings emphasize the significance of recognizing the intricate differences inherent within a minority group. Prior studies have examined acts of solidarity among varied minority groups within a single minority dimension, for example, between Black and Latine communities in the United States (i.e. stigma‐based solidarity; Craig & Richeson, 2016). However, given the multifaceted struggles tied to intersectional identities, a deeper comprehension of allyship among subsectors of minority communities, such as LGBTQIA+ communities, is essential, especially when these subgroups possess varying power dynamics due to other identity dimensions like race, ethnicity and migration status. Notably, our study illustrates how negotiations of group boundaries are crucial for those with intersectionally marginalized identities when they contemplate not just their own inclusion within queer pride but also the meaning and role of allyship. Ultimately, our findings shed light on how queer people relate to each other and how they engage in and demand solidarity both with and from one another. This emphasizes the need for understanding and fostering unity both within and between the diverse subgroups of LGBTQIA+ communities as well as the heterosexual and cisgender majority.

As our study is rooted in the Danish socio‐cultural context, it cannot be taken to represent the experiences and perspectives of LGBTQIA+ individuals in other contexts. The connection we observe between conceptualizations of allyship, on the one hand, and questions of intersectionality, on the other hand, is also a product of our decision to sample across Pride events that intersected with other identity‐based concerns around gender and race. However, we think that the intersectional power dynamics uncovered in the study, and their meaning for allyship, may hold resonance for other settings where multiple forms of oppression are entrenched within the social system. For example, the persistent risk of violence for transgender people, especially Black transgender women, in the United States (Thoreson, 2021) and the disproportionate health barriers faced by transgender women of colour (Smart et al., 2020) underscore the critical importance of focusing on intersectional dynamics when studying queer issues. In such contexts, understanding the specific needs and perspectives of those facing layered forms of oppression can contribute to more effective allyship and support.

By drawing on the lessons from these findings, we hope to inspire further research towards fostering fairer societies that genuinely recognize and address the diverse challenges of intersectional identities and oppression. Given the connection we observe between allyship and intersectionality, it would seem fruitful for research to unpick this further. Future research could, for example, examine when intersectional marginalized individuals prioritize building strength within their intersectional ingroup, when they desire support from other minority group members and when they wish to mobilize the majority outgroup. Furthermore, recognizing the significance of privileged actors within subgroups of minority communities opens new research avenues. For example, research could explore the factors that mobilize relatively privileged individuals within the minority to recognize and support more disadvantaged ingroup members. This would also have implications for advocacy efforts, which should consider the layered identities of individuals to better support and advocate for their rights.

In addition to exploring the complexities of intersectional solidarity within a minority group, it seems crucial to acknowledge instances where such solidarity is challenged or rejected, leading to fragmentation rather than solidarity within and across minority groups. Recent debates between trans activists and gender‐critical feminists, sometimes called trans‐exclusionary radical feminists, serve as a pertinent example, particularly when gender‐critical perspectives are voiced by lesbians who fear the dilution or marginalization of their unique interests and concerns within the broader LGBTQIA+ movement (Hines, 2019; Worthen, 2022). While our study did not observe these forms of intersectional hostility from relatively privileged ingroup members, we believe they necessitate examination within the broader literature on solidarity and social change.

We also think that the emergent, though still broad, three‐level conceptual framework—which highlights the multi‐layered practice of allyship, rather than allies as identifiable individuals—could be further fleshed out. At the very least, it suggests that when researchers ask questions about allyship, this should entail multiple additional questions about what exactly is done to reveal this, and at which level those actions are directed (i.e. personal, relational and/or structural). These additional nuances might help to better understand when allyship is welcomed and accepted versus questioned or rejected. Although our conceptual framework awaits such elaboration, we hope it will already aid researchers and practitioners to study and discuss allyship in a more structured and comprehensive way. Further research will be required to integrate the conceptual framework with queer people's negotiation of group boundaries to shed light on whether the engagement on different levels of allyship is expected from individuals depending on their status as a relatively privileged ingroup member or outgroup member. Regarding performative allyship, it would be valuable to further examine the motivations to engage in this form of allyship, for example, by investigating the role of paternalism and its effect on the perception of allies (e.g. Estevan‐Reina et al., 2024; Urbiola et al., 2023) in the context of LGBTQIA+ allyship.

## CONCLUSION

In conclusion, our study sheds light on the multifaceted nature of allyship within LGBTQIA+ communities, offering a structured three‐levelled framework that navigates the complexity of allyship and aims to address the conceptual ambiguity in the field. While situated in Denmark, our findings hold potential to resonate in other settings by highlighting the influence of intersectionality within minority communities and emphasizing the need to recognize and negotiate power dynamics within and between subgroups of these communities. This study underscores the role of allyship in fostering solidarity within and outside of minority groups. We hope that our conceptual framework of allyship and the analysis of dynamic group boundaries in minority communities can provide a foundation for further explorations of allyship in general and, specifically, how it should be practiced and whom it should involve. Recognizing the layered experiences of oppression within allyship efforts may foster new understandings of inequality, its manifestations and strategies for addressing it, ultimately contributing to more inclusive and impactful social change.

## AUTHOR CONTRIBUTIONS

**Bao‐Thi Van Cong:** Conceptualization; data curation; investigation; formal analysis; methodology; writing – original draft. **Séamus A. Power:** Conceptualization; project administration; supervision; writing – review and editing. **Thomas A. Morton:** Conceptualization; project administration; supervision; writing – review and editing.

## CONFLICT OF INTEREST STATEMENT

The authors report no conflict of interests.

## Data Availability

The data that support the findings of this study are available from the corresponding author upon reasonable request. The data are not publicly available due to privacy or ethical restrictions.
